# Activity Level and Type During Post-acute Stages of Concussion May Play an Important Role in Improving Symptoms Among an Active Duty Military Population

**DOI:** 10.3389/fneur.2019.00602

**Published:** 2019-06-19

**Authors:** Rosemay A. Remigio-Baker, Jason M. Bailie, Emma Gregory, Wesley R. Cole, Karen L. McCulloch, Amy Cecchini, Keith Stuessi, Taylor R. Andrews, Felicia Qashu, Lynita Mullins, Paul Sargent, Mark L. Ettenhofer

**Affiliations:** ^1^Defense and Veterans Brain Injury Center, Silver Spring, MD, United States; ^2^Naval Hospital Camp Pendleton, Camp Pendleton, CA, United States; ^3^Venesco LLC, Chantilly, VA, United States; ^4^General Dynamics Health Solutions, Silver Spring, MD, United States; ^5^Womack Army Medical Center, Fort Bragg, NC, United States; ^6^The University of North Carolina at Chapel Hill, Chapel Hill, NC, United States; ^7^Naval Medical Center San Diego, San Diego, CA, United States; ^8^Division of Program Coordination, Planning and Strategic Initiatives, National Institutes of Health, Bethesda, MD, United States; ^9^American Hospital Services Group LLC, Exton, PA, United States

**Keywords:** service members, military, concussion, mild traumatic brain injury, post-acute activity, symptoms

## Abstract

**Background:** Previous research demonstrates that early rest and gradual increases in activity after concussion can improve symptoms; however, little is known about the intensity and type of activity during post-acute time periods—specifically months post-injury—that may promote optimal recovery in an active duty service member (SM) population.

**Objective:** The objectives of this study were to investigate how activity level and type at the post-acute stages of concussion (at 1 and 3 month[s] post-injury) impact subsequent symptoms among SMs, and how this relationship might differ by the level of symptoms at the time of injury.

**Methods:** Participants included 39 SMs ages 19–44 years from 3 military installations who were enrolled within 72 h after sustaining a concussion. Linear regression was used to evaluate whether the association between activity level at 1 or 3 month(s) post-injury (as measured by a multi-domain Activity Questionnaire) and subsequent symptoms at 3 and/or 6 months (as measured by the Neurobehavioral Symptom Inventory) varied by the level of symptoms at acute stages of concussion. Partial correlation was used to evaluate relationships that did not differ by acute symptom level. Symptoms at the time of activity assessment (1 or 3 month[s]) were accounted for in all models, as well as activity level at acute stages of concussion.

**Results:** Greater physical and vestibular/balance activity at 1 month were significantly correlated with lower symptoms at 3 months, but not at 6 months post-injury. There were no significant associations found between activity (total or by type) at 3 months and symptoms at 6 months. The association between activity level at either 1 or 3 months and subsequent symptoms at 3 and/or 6 months did not differ by the level of acute symptoms.

**Conclusion:** The intensity and type of activities in which SMs engage at post-acute stages of concussion may impact symptom recovery. Although low levels of activity have been previously shown to be beneficial during the acute stage of injury, higher levels of activity may provide benefit at later stages. These findings provide support for the importance of monitoring and managing activity level beyond the acute stage of concussion.

## Introduction

Concussion, or mild traumatic brain injury, significantly impacts warfighter readiness for events such as deployment and combat. With over 380,000 diagnosed concussions among US service members (SMs) since 2000 (1), rehabilitation approaches to expedite symptom recovery after concussion are highly relevant to health and readiness in this population. A growing body of evidence has shown the importance of monitoring and regulating activity level in the first hours to days following a concussion (2–8); however, little attention has been given to the contribution of post-acute (i.e., ≥1 month[s]) activity level on symptom recovery in the weeks and months that follow. Furthermore, most of these studies have focused on sports-related concussion (2–5, 7, 8), while only a limited number provide information about active duty military personnel (9), who may have a greater risk for concussion, and among whom the consequences of persistent impairments impact military force readiness. Research has also been sparse in the evaluation of symptom recovery against activity by specific categories (e.g., cognitive, physical, vestibular/balance). This type of information could help to improve clinical guidance for primary care managers to educate and provide guidance to their patients on the most appropriate type and intensity of activities at different stages of recovery to optimize return to pre-injury activities and symptom resolution.

Activity participation, whether too little or too much, during the acute recovery period from concussion is known to impact recovery rates. Unrestricted physical activity during acute stages of concussion has been shown to negatively impact recovery (2, 4–6), and increased cognitive activity shortly following injury to lengthen recovery time (3). In contrast, studies have also demonstrated that too much rest may not provide clear benefits and may even negatively influence recovery (6, 10). In a systematic review of sports-related concussion studies, an initial period of moderate physical and cognitive rest has been shown to provide benefit during the acute post-injury phase (11). Prolonged physical and cognitive rest beyond the currently recognized recommendation by expert consensus of 2 days has also been demonstrated to be associated with higher levels of total symptoms over 10 days after injury (6, 12). Considering activity levels beyond the acute stage of injury, findings also support the importance of engagement in some activity, but an understanding of the appropriate activity level and activity type remains largely unknown. One study evaluating a cohort of student-athletes suggested that moderate physical and cognitive activity (vs. minimal activity) within 30 days of injury may be necessary for symptom recovery and better neurocognitive performance (5). In a study of concussed athletes and non-athletes, ages 16–53, with symptoms lasting at least 6 weeks post-injury but stable for 2–3 weeks, a treadmill exercise for 5–6 days per week was found to significantly decrease the level and number of symptoms after 6 weeks from measures prior to the treadmill exercise (7). Overall, these studies suggest that unrestricted activity at the acute stage of concussion may exacerbate symptoms or delay recovery; however, some level of activity in both acute and post-acute stages of concussion may be beneficial for recovery.

Regardless of outcome, existing studies have been limited to cohorts of pediatric and adult athletes (2–5, 7, 8), and findings may not be generalizable to a military population whose occupational environment differs significantly, potentially impacting the optimal types and intensities of activity during recovery. For athletes, the Consensus Statement on Concussion in Sport has recommended immediate physical and cognitive rest after a concussion, followed by a stepwise return-to-play after clearance by treating healthcare providers (13). A similar set of guidelines, the Progressive Return to Activity (PRA) Clinical Recommendation (CR), which emphasize gradual return to activity after rest at the acute stage of concussion, was developed specifically for SMs by military and civilian subject-matter experts and published by the Defense and Veterans Brain Injury Center (DVBIC) (1). Recent research among SMs suggests that activity participation within the acute stage of injury impacts symptom resolution over time (9); however, it is unknown how post-acute levels of activities, particularly the type of activities conducted to include military-specific tasks (e.g., combat training), may influence continued improvement in symptomatology.

Optimal patterns of activity during the course of concussion recovery may depend on the severity of symptoms experienced in the acute stage. In a recent study by our group, greater activity level at acute stages of concussion was associated with higher levels of symptoms over time, but only among those with high levels of acute symptoms (9). In the current study, we build upon our previous findings by evaluating the contribution of activities at later stages of concussion rehabilitation post-concussion. The primary objectives of this study were: 1) to evaluate the relationship between activity level (in total and by categories: cognitive, lifestyle, physical, vestibular/balance, and military-specific) at post-acute stages of concussion (specifically, at 1 and 3 month[s] post-injury) and subsequent symptom level among an active duty military population; and 2) to determine whether this association differs by acute symptom severity.

## Materials and Methods

Data for this study were drawn from the broader DVBIC (Defense and Veterans Brain Injury Center, RRID:SCR_004505) PRA CR Study, which investigated the impact of implementing the DVBIC PRA CR focused on gradual return to unrestricted activity post-concussion among SMs (1, 14). The parent study has been previously described in detail (15). Data from the current study included only concussed SM participants who received “usual treatment” from providers who had not yet received focused training on the PRA CR. “Usual treatment” refers to any treatment deemed appropriate by the treating clinicians; it was not experimentally controlled. This study was carried out in accordance with the recommendations of the Naval Medical Center San Diego Institutional Review Board, with concurrence from Womack Army Medical Center, and Human Research Protections Program administrative review by the Defense Health Agency. These committees approved the protocol in compliance with all applicable federal regulations governing the protection of human subjects. All subjects gave written informed consent in accordance with the Declaration of Helsinki.

### Study Participants

Participants who received “usual treatment” included 64 SMs, 18–48 years of age, recruited from clinics and operational medical units at three U.S. military installations (Army [southeast U.S.], Navy [southwest U.S.], and Marine Corps [southwest U.S.]). Eligible participants had sustained a concussion within the previous 72 h of enrollment into the study. Those who suffered other concussions within 12 months of the concussion in question were excluded. Electronic medical records were reviewed to confirm concussion diagnoses, which must have met the Veterans Administration/Department of Defense definition for concussion (16). After enrollment, participants completed a face-to-face baseline assessment (i.e., within 72 h of injury), and were followed-up at 1 week, 1 month, 3 months, and 6 months post-injury via a telephone or in-person interview.

Of the 64 participants from the parent study, there were 39 with complete data to assess activity level at 1 month and symptom level at 3 months; 38 had complete data to assess activity level at 1 month and symptom level at 6 months; and 33 had complete data to assess activity level at 3 months and symptom level at 6 months. The demographic and military characteristics of the 39 participants evaluated for activity level at 1 month and symptom level at 3 months (the greatest number of participants in any of the proposed study analyses) were comparable to those of the “usual treatment” group from the parent study consisting of 64 participants.

### Measures and Procedures

#### Assessment of Activities

This study utilized a 60-item Activity Questionnaire (9, 15), which included activities that are recommended to be avoided or encouraged at various stages of recovery according to the DVBIC PRA CR education materials (17). For each activity item, participants were asked “Since your concussion, did you [activity item stated]” during baseline and at 1 week, or, for other follow-up interviews, “In the last 2 weeks, did you [activity item stated].” Although psychometric properties are not yet available, this questionnaire was streamlined for analysis by removal of 11 items due to low variance, low correlation with other activity items, and inconsistent interpretation by SMs as demonstrated by questions asked of the interviewers during follow-up. An interdisciplinary group of investigators representing Epidemiology, Neuropsychology, Primary Care Sports Medicine and Physical Therapy (RR, JB, KS, KM, AC) reviewed the remaining 49 items and determined 6 individual categories: (1) cognitive (12 items); (2) lifestyle (10 items); (3) physical (20 items); (4) vestibular/balance (17 items); and (5) military-specific (6 items) (9). Eighteen items were included in multiple categories (e.g., walk briskly was categorized as both a physical and vestibular/balance activity). To ensure that scores represented the same direction, seven items (e.g., sleep 6–8 h a night) were reverse coded. Three items did not fit into any category (“wear dark glasses or sunglasses” and “do familiar tasks [e.g., vehicle maintenance check],” “rest all day”), but were included in the total activity score. Each item was scored 0–4 with responses such as “never” (0), “every few days” (1), “some days” (2), “most days” (3), and “every day” (4).

#### Assessment of Neurobehavioral Symptoms

The Neurobehavioral Symptom Inventory (NSI) was used to assess concussion symptoms (18). Participants were asked to evaluate 22 symptom items since their concussion (during baseline and at 1 week), or, in the last 2 weeks, during the 1-, 3-, and 6-month follow-up interviews. Based on previous exploratory analyses, these items were categorized into 4 factors: (1) cognitive (4 items); (2) vestibular (3 items); (3) somatosensory (7 items); and (4) affective (6 items) (19, 20). Two items regarding hearing difficulty and changes in appetite were only included in the total symptom score as they did not fit into any of the 4 factors. Responses ranged from 0 to 4 and included “none” (0), “mild” (1), “moderate” (2), “severe” (3), and “very severe” (4). Among SMs, NSI has been shown to have high internal consistency (total alpha = 0.95; subscale alpha = 0.88–0.92) (21). Further, external validity was exemplified with moderate correlation (*r* = 0.41) showing NSI differentiating veterans with TBI status from those without (21).

#### Calculation of Activity and Symptom Scores

To maximize the use of available data, a prorated summary score was calculated for activity and symptom level for participants with at least one missing item needed to calculate each variable (22). All non-missing values per variable were summed and multiplied by the total number of possible items. This, in turn, was divided by the actual number of items with non-missing values. For each category of activity or symptom, prorated scores were based on the items included within each category. A list of items within each category has been reported previously (9). In the current study, only one participant had 1 missing item for the calculation of activity level. Prorated scores were transformed into z-scores for comparability across categories. Each mean and standard deviation were based on the type of activity or symptom level in question. For example, activity level was assessed as a total score and by categories such as cognitive activity. To calculate the z-score for total score, this took into consideration the mean and standard deviation of all of the activity item responses from all participants. To calculate the z-score for cognitive activity, this took into consideration the mean and standard deviation of activity items that were categorized as cognitive activity, also from all participants. All of these calculations were done at each time point.

#### Assessment of Covariates

Demographic characteristics (i.e., age, sex, and education) and military information (i.e., branch affiliation, current rank, and number of deployments) were assessed at baseline.

### Statistical Analyses

To ascertain the contribution of demographic characteristics and military history on activity level during post-acute stages of symptom recovery, Spearman correlation was used for age, a continuous variable, and one-way analyses of variance for categorical variables. The modifying impact of symptom level at the acute stage of concussion was also analyzed and presented as a dichotomized variable (high vs. low) based on median cut-offs (total and by categories). Although evaluating this variable continuously would provide more statistical power, dichotomization was chosen to enhance clinical relevance. First-order interaction terms were created as a product of this variable with post-acute activity level. In these investigations, linear regression was utilized to assess the relationship between activity level at either 1 or 3 month(s) and subsequent symptoms (i.e., at 3 and 6 months, or at 6 months, respectively) among those with either high vs. low level of symptoms at the acute stage of injury (objective #2). Partial correlation (r_*p*_) was used to evaluate relationships that did not differ by acute symptoms (objective #1). All models were adjusted for activity level within 72 h of injury and symptom level at the time of each activity variable being examined. To investigate assumptions for the utility of linear regression and Pearson correlation in our study, we used the following techniques: to identify non-linearities in the data, we used scatter plots and graphs of augmented component-plus-residual plots with LOWESS smoothing; to assess the assumption of normality, we evaluated graphs of standardized normal probability plots (P-P plots) to assess non-normality in the middle range of data, and quantiles of the residual against quantiles of a normal distribution (Q-Q plots) to assess non-normality at the tail-ends of data; to test the assumption of homoscedasticity, a plot of residuals vs. fitted (predicted) values were examined; and to evaluate evidence of auto-correlation, we utilized the Durbin-Watson statistic. All assessments were done between each activity level score (total and by categories) against each symptom level score (total and by categories). To address the presence of outliers that may significantly affect results, both the activity and symptom level data (z-scores) were truncated to +/−3 standard deviation from the mean.

Main effects and interaction were considered significant at a *p* < 0.05. All statistical analyses were completed using Stata statistical software (Stata, RRID:SCR_012763), release 15 (StataCorp, 2017, College Station, TX).

## Results

**Figure 1:**
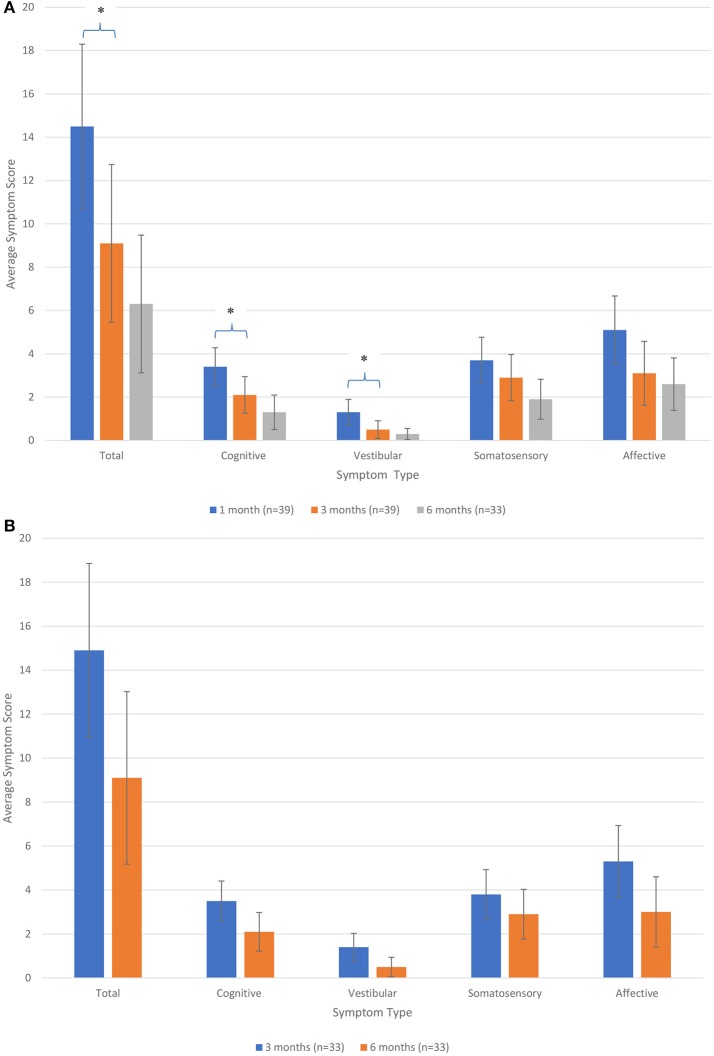
**(A)** Average symptom level score over time (in total and by categories) for participants assessed for activity level at 1 month and symptom level at 3 months post-injury. ^*^Significant at *p* < 0.05. **(B)** Average symptom level score over time (in total and by categories) for participants assessed for activity level at 3 months and symptom level at 6 months post-injury.

There was an overall decrease in symptom progression (total and by categories) over time (see Figures 1A,**B**). Among the 39 participants evaluated for activity level at 1 month and symptom level at 3 months, a significant decrease in total, cognitive and vestibular symptoms were found from 1 month to 3 months post-injury. Although the reduction continues to 6 months, the progression was not statistically significant. See Figure 1A. Among the 33 participants evaluated for activity level at 3 months and symptom level at 6 months, the decrease in symptom progression (total and by categories) was not statistically significant (see Figure 1B).

Table 1 illustrates the characteristics of the study sample. Participants had a mean age of 26 years (range = 19–41) and were mostly men (89.7%), married (46.0%), and had 12–15 years of education (82.0%). Most served in the US Army (69.2%), were non-commissioned officers (E4 and E5, 51.3%) and were deployed at least once (56.4%). There were no significant differences in total activity or symptom score at 3 months post-injury by any assessed demographic or military characteristic. Study participants had a median symptom score of 20 (range = 2-61) at the acute stage of concussion.

**Table 1:** Demographic and military characteristics of service members and their relation to activity and symptom score at 3 months post-injury, overall (*n* = 39).

**Variable**	**Value**	**Total activity score**		**Total symptom score**	
		**Mean (SD)**	***p***	**Mean (SD)**	***p***
Age in years, mean (SD)	26 (6.2) (Range = 19–41)	NA	0.938	NA	0.393
Sex, *n* (%)			0.804		0.283
Men	35 (89.7)	107.3 (19.6)		15.2 (12.5)	
Women	4 (10.3)	109.8 (8.5)		8.3 (5.3)	
Education, *n* (%)			0.754		0.624
High school diploma or GED	13 (33.3)	103.6 (14.1)		12.3 (9.0)	
Some college (1–3 years)	19 (48.7)	111.2 (22.5)		16.1 (13.5)	
College graduate (4+ years)	4 (10.3)	103.0 (18.8)		19.5 (16.3)	
Some graduate school	1 (2.6)	118.0 (NA)		8.0 (NA)	
Graduate/professional program	2 (5.1)	102.0 (2.8)		6.0 (7.1)	
Marital status, *n* (%)^a^			0.821		0.496
Never married	15 (40.5)	104.3 (19.2)		17.4 (14.4)	
Married	17 (46.0)	107.4 (20.3)		13.9 (10.8)	
Divorced	4 (10.8)	114.5 (15.2)		10.5 (11.1)	
Separated	1 (2.7)	104.0 (NA)		1.0 (NA)	
Branch, *n* (%)			0.102		0.351
US Navy	3 (7.7)	96.3 (13.3)		5.7 (8.1)	
US Marine Corp	9 (23.1)	118.3 (13.7)		17.4 (11.6)	
US Army	27 (69.2)	105.2 (19.4)		14.4 (12.4)	
Rank, *n* (%)			0.870		0.883
Junior enlisted (E1-E3)	8 (20.5)	108.8 (15.9)		15.8 (10.0)	
Non-commissioned officer (E4-E5)	20 (51.3)	103.4 (19.7)		13.7 (13.0)	
Non-commissioned officer staff (E6-E9)	8 (20.5)	102.6 (22.9)		16.5 (14.6)	
Officers (O1-O10)	3 (7.7)	111.3 (9.9)		10.7 (2.5)	
Number of deployments, *n* (%)			0.895		0.211
0	17 (43.6)	107.1 (20.1)		17.2 (9.8)	
1+	22 (56.4)	108.9 (18.0)		12.3 (13.4)	
Acute symptom score, median (IQR)	20 (15–37) (Range = 2–61)	NA	NA	NA	NA

*IQR = Interquartile Range*.

a *n = 37 for this variable*.

### Activity Level at 1 Month and Subsequent Symptoms at 3 and 6 Months

The association between activity level at 1 month (total or by categories) and subsequent symptoms at 3 and 6 months did not differ by the level of symptoms within 72 h of injury. Table 2 provides the correlations between activity level at 1 month and symptom level at 3 and 6 months post-injury. Greater total activity at 1 month post-injury was significantly correlated with lower cognitive (r_*p*_ = −0.41, *p* = 0.012) and somatosensory (r_*p*_ = −0.36, *p* = 0.028) symptoms at 3 months. Subscale analyses suggested that total score correlations were driven primarily by physical and vestibular/balance activities. Greater physical activity at 1 month was significantly correlated with lower cognitive (r_*p* =_−0.44, *p* = 0.006) and vestibular (r_*p* =_−0.37, *p* = 0.025) symptoms at 3 months post-injury. Greater vestibular/balance activity at 1 month was also significantly correlated with lower total (r_*p* =_−0.39, *p* = 0.017) and cognitive (r_*p* =_−0.55, *p* < 0.001) symptoms at 3 months post-injury. No significant correlations were found between total, cognitive, lifestyle and military-specific activities at 1 month and symptoms (total or by category) at either 3 or 6 months.

**Table 2:** Partial correlation between activity level at 1 month (total and by categories) and symptom level at 3 and 6 months (total and by categories), adjusted for activities at acute stage of concussion and symptoms at 1 month post-injury.

**Symptom level months post-injury**	**Activity level at 1 month post-injury, correlation coefficient (*****p*****-value)**					
	**Total**	**Cognitive**	**Lifestyle**	**Physical**	**Vestibular/balance**	**Military- specific**
**3 MONTHS (*****n*** **=** **39)**						
Total score	−0.30 (0.070)	0.002 (0.992)	−0.06 (0.730)	−0.30 (0.068)	−0.39 (0.017)^*^	−0.07 (0.661)
Cognitive	−0.41 (0.012)^*^	−0.08 (0.622)	0.03 (0.868)	−0.44 (0.006)^*^	−0.55 (<0.001)^*^	−0.14 (0.423)
Vestibular	−0.17 (0.310)	0.13 (0.451)	0.18 (0.288)	−0.36 (0.026)^*^	−0.31 (0.066)	0.09 (0.606)
Somatosensory	−0.36 (0.028)^*^	−0.07 (0.683)	−0.17 (0.327)	−0.30 (0.076)	−0.30 (0.067)	−0.21 (0.203)
Affective	−0.17 (0.312)	0.03 (0.852)	0.03 (0.880)	−0.19 (0.270)	−0.30 (0.067)	−0.03 (0.847)
**6 Months (*****n*** **=** **38)**						
Total score	−0.11 (0.506)	0.06 (0.731)	−0.06 (0.727)	−0.19 (0.275)	−0.16 (0.343)	0.09 (0.617)
Cognitive	−0.21 (0.227)	0.10 (0.558)	0.08 (0.663)	−0.31 (0.064)	−0.30 (0.080)	−0.02 (0.893)
Vestibular	−0.15 (0.370)	0.10 (0.567)	−0.07 (0.695)	−0.28 (0.101)	−0.17 (0.319)	0.05 (0.761)
Somatosensory	−0.07 (0.667)	0.11 (0.514)	−0.04 (0.822)	−0.14 (0.411)	−0.13 (0.460)	0.08 (0.661)
Affective	−0.10 (0.576)	−0.10 (0.550)	−0.11 (0.535)	−0.12 (0.500)	−0.10 (0.560)	0.09 (0.585)

* *Significant p-value at a level < 0.05 (bolded)*.

### Activity Level at 3 Months and Subsequent Symptoms at 6 Months

The relationships between activity level at 3 months and subsequent symptoms at 6 months post-injury did not vary by the level of symptoms within 72 h of injury. Further, no significant relationships were found between activity level at 3 months (total or by categories) and symptoms at 6 months post-injury (data not shown).

## Discussion

This study provided preliminary evidence that activity level at post-acute stages of concussion may play an important role in improving symptoms among SMs; specifically, SMs with higher activity levels at 1-month post-injury reported lower level of post-concussion symptoms 2 months later. These findings held even after controlling for severity of symptoms at 1 month and activity level shortly after injury, suggesting that the effects of post-acute activity levels on later symptom outcomes were not simply due to pre-existing differences in trajectory of recovery. The results of this study suggest that although low levels of activity have been previously shown to be beneficial during the acute stages of injury among SMs (9), higher levels of activity may provide benefit at later stages in this population. The effects of activity level on later symptoms appeared to level off after approximately 3 months, possibly due to a greater proportion of SMs having achieved successful symptom recovery around this time. As we reported previously in data drawn from the parent study (9), only 26.8% of SMs demonstrated clinically significant levels of post-concussive symptoms by 3 months post-injury.

Of interest, the type of activity was important to symptom resolution; specifically, higher rates of physical and vestibular/balance activities at 1 month were associated with reduced total, cognitive, and vestibular symptoms at 3 months. In contrast, cognitive, lifestyle and military-specific activities at 1 month were not associated with symptoms at 3 months. These findings were consistent with our previous study evaluating the impact of early post-injury activity, in which both physical and vestibular/balance activities were also shown to drive the relationship between activity level and symptoms over time (9). The current study did not find the association between activities at post-acute stages of concussion and subsequent symptoms to vary by the level of acute symptoms; however, other factors such as lifetime history of concussion could modify this relationship, as symptoms from previous injury may lower cerebral reserve that would otherwise be used for symptom recovery related to the current concussion. Future studies that evaluate the contribution of lifetime concussion history on symptom recovery among concussed SMs are warranted. The findings of this study demonstrate the importance of primary care managers to monitor specific types of activity for symptom recovery at the post-acute stages of concussion, as focusing on activity in total may overlook the specific impact of physical and vestibular/balance activities on symptom resolution. This could, in turn, lead to poor recovery. The preliminary findings from this paper also support exploration of rehabilitation programming/clinical care that includes physical and vestibular/balance activities, notably at chronic stages of concussion.

A limited number of studies have evaluated the contribution of activities at the post-acute stages of concussion on improving symptoms (7), and none among an active duty military population. Aside from our recent work examining acute activities (9), to our knowledge, no previous studies have considered multiple activity categories, including military-specific items, at a granular level as investigated in the current analyses. The PRA CR developed by DVBIC, from which the activities assessed in this study are obtained, provides an algorithm for activity progression, which can be standardized across military populations as a part of acute and post-acute concussion management (17). The use of this CR may improve outcomes by providing guidance to primary care managers, particularly in reengaging in physical and/or vestibular/balance activities as supported by our preliminary findings, to optimize the speed of recovery. This may not only guide the prescription of specific patterns of activity, but also, the determination of whether patients should receive medical waivers from engaging in usual duties. Future studies will include the evaluation of activity progression on improving symptom levels, comparing those who receive usual care to those who receive care according to the guidelines published in the DVBIC PRA CR.

The strengths of this study included the use of longitudinal activity and symptom data that extend 6 months post-injury within a population of military SMs. This study also evaluated demographic and military service information to assess for potential confounding factors. Additionally, data on activity items and categorization specific to a military population were collected to best address the objectives of this study. There were also limitations to consider. Although activity level was evaluated against symptom level at a later time point, controlling for symptoms at the time of activity assessment to support directionality of the association, we cannot infer causality in the significant relationships found. In addition, the Activities Questionnaire used in this study was developed *de novo* in order to be consistent with the recommendations found in the PRA CR education materials. It should be noted, however, that careful item screening was used to narrow the final list of items used in analyses. Further, this questionnaire not only included military-specific activities, but it also allowed for activity categorization not queried in previously studied and/or published surveys, most of which were aimed to evaluate a different population of individuals with moderate to severe traumatic brain injury. Along with activity level, the level of symptoms was self-reported which may be subject to recall bias. Although self-reported instruments are time- and cost-effective, future studies utilizing more objective assessment of activity (e.g., wearable devices, clinically-monitored activity, and symptom level) might help to validate the results of our findings. Finally, sample size was limited and, in part due to participant attrition over time, conservative corrections for multiple comparisons were not feasible in this study. However, even with a limited sample size, significant results were found. Nonetheless, the findings in this study were preliminary and analyses were exploratory. Future longitudinal studies with a larger sample size may be necessary not only to confirm the findings in this study, but also, to potentially detect significant effects that may have been missed in this study due to limited sample size. Additionally, with a larger sample, the trajectories of the residuals in the relationship between activity and symptom level might be better defined and might perhaps suggest the use of a more complex analytic approach.

Our findings provide preliminary evidence that activity level during the post-acute stages of concussion remains an important factor in improving symptoms, thus, advocating for continued monitoring and patient management even up to 1 month post-injury. Taken together with our recent finding that greater activity levels immediately following injury (i.e., within 72 h) result in poorer symptom status among SMs (9), these findings support recommendations for a gradual increase in activity (1, 10, 13, 14, 23–26)—rather than extended rest—to promote symptom resolution among SMs with recent concussion. Also consistent with our previous work (9), the results highlight the importance of physical and vestibular/balance activities in managing symptoms over time. Further research is necessary to evaluate the mechanisms of these associations and potential differences in the impact of various types of activities on symptom resolution. Future studies that evaluate how changes in activity level over time influence symptom recovery will also be critical to the continued development of clinical protocols for improving health outcomes of military personnel who have been diagnosed with concussion. Cumulative findings may inform how primary care managers and rehabilitation providers educate and guide their patients regarding progressive return to activity to optimize outcomes and military readiness.

## Data Availability

The datasets analyzed for this study are not publicly available but will be submitted to FITBIR beginning in 2019. Requests to access the datasets should be directed to Dr. Rosemay Remigio-Baker (rosemay.a.remigio-baker.ctr@mail.mil).

## Ethics Statement

This study was carried out in accordance with the recommendations of the Naval Medical Center San Diego Institutional Review Board, with concurrence from Womack Army Medical Center, and Human Research Protections Program administrative review by the Defense Health Agency. These committees approved the protocol in compliance with all applicable federal regulations governing the protection of human subjects. All subjects gave written informed consent in accordance with the Declaration of Helsinki.

## Author Contributions

RR-B, ME, JB, EG, and WC were involved in the development of concept and analytical approach. All analyses were conducted by RR-B. Screening and categorization of activity items used in the study were done by EG, RR-B, JB, KS, KM, and AC. KM and AC served as subject-matter experts regarding activity involvement during concussion recovery among service members. KS served as a topic expert on TBI and education, particularly as it pertains to the Progressive Return to Activity Clinical Recommendation. AC and TA were involved in data collection and provided information regarding study procedures and interaction with participants to interpret data. EG and FQ provided knowledge in the structure and content of the parent study that served as the source of data for this investigation. KS, PS, and LM provided insight into military physical and psychiatric environment. All authors contributed to the development and editing of the manuscript.

### Conflict of Interest Statement

RR-B was employed by Venesco LLC. JB and KS were employed by the General Dynamics Health Solutions. ME was employed by the American Hospital Services Group LLC. The remaining authors declare that the research was conducted in the absence of any commercial or financial relationships that could be construed as a potential conflict of interest.

## Abbreviations

SM: Service Members
DVBIC: Defense and Veterans Brain Injury Center
PRA: Progressive Return to Activity
CR: Clinical Recommendation.
